# Librarians collaborating with instructors for course integration of virtual reality

**DOI:** 10.5195/jmla.2025.2090

**Published:** 2025-07-01

**Authors:** Ryn Gagen, Brooke Olson, Merete Christianson, Nicole Theis-Mahon

**Affiliations:** 1 ryngagen@umn.edu, Health Sciences Library, University of Minnesota, Minneapolis, MN; 2 bolson16@umn.edu, Health Sciences Library, University of Minnesota, Minneapolis, MN; 3 merete@umn.edu, Health Sciences Library, University of Minnesota, Minneapolis, MN; 4 theis025@umn.edu, Health Sciences Library, University of Minnesota, Minneapolis, MN

**Keywords:** Libraries, Medical Education, Virtual Reality, Instruction, Librarians

## Abstract

**Background:**

Health science libraries have invested in virtual reality technology and spaces to support use of this technology for teaching, learning, and research. Virtual reality has many uses within health sciences education such as simulation, exploration and learning, and soft skills development. It can also be used to build empathy in health sciences students through applications that provide an immersive, first-person perspective.

**Case Presentation:**

This case describes how a health sciences library and liaison librarians partnered with a course instructor to support a class utilizing the library's virtual reality resources. Librarians were collaborators in the development of the class and facilitated class sessions in the Virtual Reality Studio. Class sessions utilized the Beatriz Lab by Embodied Labs to increase empathy in medical students who were interested in working with geriatric or Alzheimer's patients.

**Conclusion:**

Liaison librarians support teaching and learning through a variety of tools and resources, including virtual reality. By partnering with instructors, librarians can use their instruction and collection knowledge to design and facilitate classes that are meaningful and interactive. Virtual reality applications provide another resource that librarians can incorporate into their course-integrated instruction sessions.

## BACKGROUND

Virtual reality (VR) is an immersive technology that provides unique opportunities for learning through the simulation of being physically present in computer-generated environments with realistic sensory experiences. The 2016 Horizon Report identified virtual reality as an emerging trend revolutionizing medical education, allowing students to visualize and interact with complex real-world data, train in adaptive immersive scenarios, and receive real-time feedback [1]. Today, VR is utilized in a variety of ways in medical education, such as augmenting anatomy instruction [2], teaching surgical training techniques [3], and enhancing empathy [4].

Virtual reality has potentially promising means for facilitating experiential learning, an area of emphasis for many academic libraries and in the health sciences [5]. Over the last two decades, libraries have increasingly transformed their role from repositories of information into centers for the creation of new knowledge [6]. As a result, many academic libraries made investments in VR over the last decade, continuing their role of providing access to new technology [7]. VR's educational potential has been particularly prominent in the health sciences, prompting libraries to acquire VR applications for health sciences education [8,9]. Immersion in virtually simulated scenarios allows students to experience the mindset and perceptions of patients by engaging users in sensory experiences that simulate the lived realities of medical conditions. For example, virtual reality's use as a perspective-taking educational tool has shown a demonstrated impact on empathy for people living with dementia [10].

VR simulations of patient experiences create unique opportunities for medical students to practice competencies and build empathy unavailable in other forms of training. For example, many medical school simulation programs include standardized patients, or actors, to play the part of a patient in medical scenarios while the students play the role of the health care provider. While this is important training, it does not put medical students in the shoes of the patient to help them understand the patient's lived experiences. The first-person perspective of wearing a VR headset can strongly influence students' affective response to patients with dementia through simulating disease symptoms, sensory distortions, internal narratives, and social frustration. This helps students be more conscious of how they interact with patients and their caregivers due to a firsthand understanding of how they might be feeling. As a result, students may emerge from a VR learning experience more confident in making informed person-centered clinical decisions [10].

This case report contributes to the ongoing discussion of VR as an educational resource and instructional tool within medical librarianship, describing how librarians at the University of Minnesota Health Sciences Library partnered with a geriatrics instructor on course-integrated virtual reality instruction sessions.

## CASE PRESENTATION

In Fall of 2023, the University of Minnesota Medical School launched a new curriculum for medical students. Becoming a Doctor (BaDr) is a new series of classes woven throughout the undergraduate medical curriculum that empowers students as they transition into practicing medicine. BaDr has four learning objectives: professional identity formation, reflective practice, clinical skill advancement, and community building. One week per semester is dedicated exclusively to BaDr classes, with a mix of required and elective class sessions that address one or more of the learning objectives. Methods of instruction include lectures, discussions, case-based learning, and simulations. Sessions are standalone classes that require all student work to be completed within the session's allotted time. Instructors cannot include pre-work or homework, and assessments or evaluations must also occur during class time.

Instructors interested in creating and leading a BaDr class submit proposals describing the audience, facilitators, content, and class learning activities. BaDr course directors review and accept proposals and assist in the administration of approved class sessions. Librarians have partnered with faculty and co-developed several BaDr classes. Examples of previous partnerships include a class on evidence-based clinical information and a class on integrating traditional health knowledge in oral histories into clinical practice. Once approved, course instructors fully develop their proposals, including finalizing titles, descriptions, and learning objectives; designing instructional content and active learning activities; and recruiting additional facilitators.

**Figure 1:**
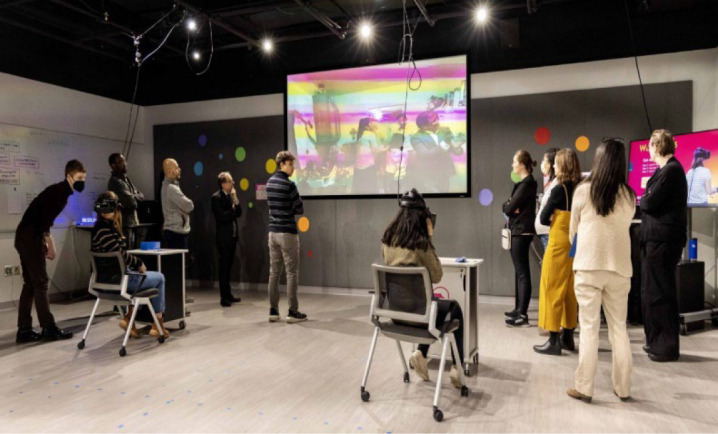
Class in VR Studio

The Health Sciences Library (HSL) at the University of Minnesota moved to a new building in 2021. The newly relocated library supports multiple spaces for emerging technology, including a Virtual Reality Studio (VR Studio) (Figure 1). One role of the VR Studio is to support teaching, learning, and education around immersive technology in the health sciences. In addition to the VR Studio space, HSL also received an allocation to acquire and license virtual reality applications. As a result, HSL licensed Embodied Labs [11], a VR application which provides first-person perspective simulations. One of the labs included in HSL's subscription is the Beatriz Lab, which allows users to embody the perspective of Beatriz, a middle-aged Latina woman diagnosed with Alzheimer's disease. The lab progresses through three modules that reflect the stages of Beatriz's Alzheimer's: early-stage, mid-stage, and late-stage (Table 1). Learners gain insights into her daily struggles, internal thoughts, interpersonal relationships, and sensory experiences — including alterations in visual and auditory processing. Through witnessing Beatriz's sensory and cognitive changes, along with her struggles to communicate her confusion to her family, the VR simulation highlights what individuals with dementia may experience. In summer 2023, an instructor specializing in geriatric education contacted HSL about using the Beatriz Lab for a BaDr class session proposal. The instructor was interested in VR as a tool for first-person simulation and enhancing student learning about dementia.

**Table 1:** Embodied Labs Beatriz Lab Content

Module Title and Runtime	Experiential and Interactive Components
Module 1: Early-Stage Alzheimer's (8:00 minutes)	Experiential components: Confusion, memory issues, and loss of cognitive function at work. Difficulty recognizing and engaging in interpersonal communication with family members, such as children and grandchildren. Disorientation and loss of independence while grocery shopping. Inability to carry out familiar, multi-step tasks such as cooking and planning for family celebrations. Changes to sensory and emotional processing, such as distortion of voices and inner monologue demonstrating increased worry and frustration. Interactive components for users: Prompted to respond as Beatriz in conversations. Interact with objects while grocery shopping or preparing dinner.
Module 2: Mid-Stage Alzheimer's (11:00 minutes)	Experiential components: Appearance of sundowning symptoms, including distortion of audiovisual processing and significantly increased fear. Beatriz perceives a visiting family member as a home intruder. Beatriz loses independence and now lives with her daughter and grandson. Beatriz becomes disoriented and falls while showering, demonstrating functional loss surrounding self-care tasks. Beatriz participates in a family care plan meeting, but feels isolated and confused when she is unable to understand the discussion due to mishearing words and not understanding why home health care professionals are present. Beatriz's inner thoughts become more emotionally intense and disoriented. Interactive components for users: Engage in a coloring activity during the family meeting.
Module 3: Late-Stage Alzheimer's (4:15 minutes)	Experiential components: Beatriz receives care from a certified nurse assistant. Beatriz experiences significant audiovisual disturbances during a family Christmas celebration. Beatriz experiences some reduction in symptom severity while familiar music plays. Interactive components for users: None

This BaDr proposal was accepted in November 2023 and the class sessions were offered in early January 2024. The overall objective of the class was for students to engage in a meaningful immersive experience and reflect on their clinical approach to individuals with dementia. Two identical sessions were offered at separate times on the same day to third- and fourth-year medical students with an enrollment cap of twenty students per session. Enrollment was limited to ensure all students had sufficient time to engage with the Beatriz Lab in the VR Studio.

The students began the ninety-minute class as a large group, and after an introduction from the instructor, they completed the Dementia Attitudes Scale as a pre-assessment [12]. Next students were divided into two groups of ten students, with one group remaining in the classroom for a small group exercise led by the instructor, while the other group completed the Beatriz Lab facilitated by librarians in the VR Studio. Each group switched locations halfway through the class to give students an equal amount of time in the VR Studio and the classroom.

The Beatriz Lab was divided into segments of equal duration, so that each individual student could experience immersion in the VR headset for one to two scenes within each module of the Beatriz Lab. These scenes showed the progression of her Alzheimer's Disease. Two synchronized groups of students engaged with the VR content simultaneously in the VR Studio so that students who were not wearing the VR headset could watch their classmates' experiences in the Beatriz Lab projected on a large screen in the VR Studio.

Two clinical geriatricians facilitated small group discussions in the VR Studio, prompting students to think about how they would interact with a patient like Beatriz and making connections between the content shown in the Beatriz Lab and their clinical experiences. One facilitator, reflecting on the content of Module 3, commented on how music had a profound effect on Beatriz and triggered memories, even though she was in the late stage of Alzheimer's. Students actively engaged in these brief facilitated discussions with the geriatricians and learned from the geriatricians' clinical experiences. Formative assessment of student learning took place during these conversations, in which instructors were able to gauge student understanding of patient experiences of Alzheimer's and students had the opportunity to ask clarifying questions as needed.

**Figure 2:**
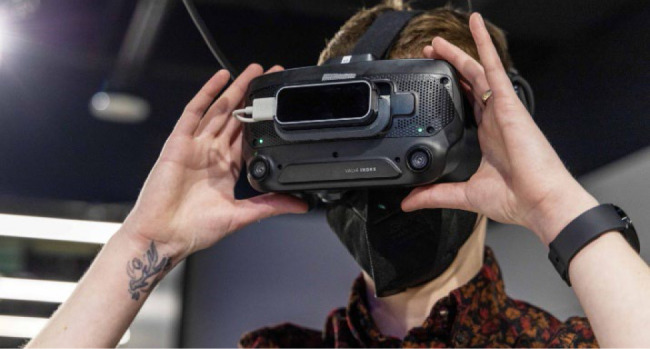
Valve Index Headset with Leap Motion Controller

Students adapted quickly to the use of virtual reality for the Beatriz Lab, especially as they watched their classmates through a screencast of the Lab as it progressed. While most students immersed in virtual reality spent time looking around and experiencing the perspective of Beatriz, some needed reminders that they could use hand tracking controls to interact with some of the features. Because Embodied Labs utilizes Leap Motion Controllers (Figure 2) mounted to the front of the headsets, students did not have to hold handheld controllers. This increases realistic immersion but is not as intuitive unless students lift their hands high enough to see the virtual representation of their hands in the VR application. Students also needed prompting to understand voice recognition cues within the Lab. Despite this, students remained focused and immersed in the Lab content whether or not they were actively wearing the headset.

The virtual reality experience was accompanied by a classroom activity led by an instructor who asked students to cut paper into twenty-five pieces [13]. On these pieces of paper, they were asked to write five things they love, five of the most important people in their life, five activities they enjoy, five tasks of daily living, and five places that are important to them. Students were then asked to organize their pieces of paper and lay them out in front of them. The instructor led the class through a scenario where they needed to give up the pieces of paper in response to various prompts guiding students through a process of forgetting and loss. The purpose of this exercise was to build empathy through a self-reflective process. Throughout the session students were quiet and as the activity progressed many of them had downcast expressions or put their hands on their faces. At the end of the exercise many students expressed emotions of sadness and frustration, with some hoping that they would not have to give everything up.

At the end of the class, students reconvened as a larger group to debrief with the geriatrician facilitators and instructor about their experience with the Beatriz Lab and the classroom activity. Students shared that they experienced frustration, lack of authority in decision making, loss, and sadness in both activities. Facilitators shared approaches from their own clinical experiences such as the importance of slowing down so patients can take in information, the frustration that caregivers and patients may experience, and how utilizing tools like music can help connect with patients with dementia.

## DISCUSSION

Through VR class sessions, librarians witnessed firsthand how medical students engaged with the VR Studio and licensed applications. Observations revealed that students' experiences may have been influenced by limited prior familiarity with VR. When asked at the beginning of the class how familiar they were with VR, most of the students indicated this was their first experience. This case presented an opportunity to make an impact on the students by introducing them to VR, and although they adapted quickly during the session, there were some logistical hurdles in orienting students to the technology. No previous knowledge of VR was assumed when planning these class sessions, so each group was provided with a brief overview of how to wear and adjust the headsets and use the Leap Motion Controllers. Despite this initial orientation, some students did not interact with the Lab in the absence of handheld controllers. Students may not have had adequate time to acclimate to the technology, which may have limited their confidence or level of interaction with the Lab. Encouraging students to come into the VR Studio's open hours to familiarize themselves with VR before class sessions may result in a more effective learning experience in the future, especially in time-bound class sessions like BaDr that do not permit pre-work or homework [14].

Opportunities for students to orient and familiarize themselves with VR environments are important, but environmental and technical limitations can also influence the utilization of VR for learning. For example, as wearable technology, the design of VR headsets and controllers can impact user experiences with the applications. Embodied Labs utilizes hand tracking through Leap Motion Controllers which limits HSL to using Valve Index headsets that require a tethered cable connection to a compatible computer. This limited the full experience of Embodied Labs to our VR Studio and dedicated workstations within that space. Understanding the nuances, configuration, and technical requirements of VR hardware and applications can be difficult. Managing the setup and maintenance of a VR space requires constant attention and time to test that the necessary software is running [15]. Institutional settings, information technology (IT) and cybersecurity requirements, and other policies can also impact the utility of VR in educational settings. Fortunately, the HSL VR Studio has a full-time academic technologist that manages the equipment and ensures that applications are up to date.

Unfortunately, the HSL VR Studio does not have the resources to independently develop applications for educational use, resulting in a reliance on commercially available applications. Trialing commercially available VR applications is one option libraries have to identify which products will be valuable to their users; however, relying on commercial products necessitates an understanding of hardware compatibility requirements [15]. Library staff supporting VR may need to understand how to operate and support headsets manufactured by multiple brands. Reliance on commercial products can also present challenges with vendor updates and institutionally mandated IT updates and restrictions. This may render VR applications temporarily unusable until vendor or institutional IT support can assist in troubleshooting. Despite these limitations, commercial products present a solution for libraries interested in providing access to VR applications. As with any item added to a library's collection, librarians need to understand what the content is, how users can access it, and how to contact vendors for customer support if necessary. In the case of VR, this means developing an understanding of the types of VR headsets available, determining compatibility between headsets and applications, and learning how to navigate both the hardware and software interfaces for maintenance and troubleshooting.

Libraries, including HSL, acquired VR equipment and applications with the goal of integrating VR into curricula [14]. Libraries have demonstrated potential as facilitators for VR technology and application access. In addition to being facilitators for access, the role of librarians as instructional collaborators creates rich partnerships in educational applications of VR technology. Librarians bring expertise in information literacy and library resources to develop impactful course content [16]. Furthermore, students' perceptions of librarians have changed over the years to reflect the expansion and evolving roles of librarians as teachers [17]. Successful course-integrated experiences with librarians develops relationships with instructors, increases how embedded librarians are in curricula, creates positive testimonials, and fosters future collaboration [18]. Interacting with librarians in an immersive educational setting may help reduce student apprehension about reaching out to their librarians and utilizing library services [19]. After the Embodied Labs class sessions, librarians encouraged medical students to reach out and visit the VR Studio during open hours. The inclusion of librarians in course-integrated instruction and the exposure of students to technology-rich library spaces is an opportunity to invite both instructors and students to learn more about the library and connect with librarians in the future.

While libraries can play a critical role in providing access to VR equipment and applications, their potential for instructional involvement and integration goes deeper. Librarians have long been important collaborators in instruction by offering a variety of resources that include media, databases, books, and journals. Virtual reality is another medium wherein librarian expertise and knowledge of information literacy can be employed for successful course-integrated instruction.

## Data Availability

There is no data associated with this article.
